# Current recommendations for procedure selection in class I and II obesity developed by an expert modified Delphi consensus

**DOI:** 10.1038/s41598-024-54141-6

**Published:** 2024-02-11

**Authors:** Mohammad Kermansaravi, Sonja Chiappetta, Chetan Parmar, Scott A. Shikora, Gerhard Prager, Teresa LaMasters, Jaime Ponce, Lilian Kow, Abdelrahman Nimeri, Shanu N. Kothari, Edo Aarts, Syed Imran Abbas, Ahmad Aly, Ali Aminian, Ahmad Bashir, Estuardo Behrens, Helmuth Billy, Miguel A. Carbajo, Benjamin Clapp, Jean-Marc Chevallier, Ricardo V. Cohen, Jerome Dargent, Bruno Dillemans, Silvia L. Faria, Manoel Galvao Neto, Pierre Y. Garneau, Khaled Gawdat, Ashraf Haddad, Mohamad Hayssam ElFawal, Kelvin Higa, Jaques Himpens, Farah Husain, Matthew M. Hutter, Kazunori Kasama, Radwan Kassir, Amir Khan, Mousa Khoursheed, Matthew Kroh, Marina S. Kurian, Wei-Jei Lee, Ken Loi, Kamal Mahawar, Corrigan L. McBride, Hazem Almomani, John Melissas, Karl Miller, Monali Misra, Mario Musella, C. Joe Northup, Mary O’Kane, Pavlos K. Papasavas, Mariano Palermo, Richard M. Peterson, Ralph Peterli, Luis Poggi, Janey S. A. Pratt, Aayad Alqahtani, Almino C. Ramos, Karl Rheinwalt, Rui Ribeiro, Ann M. Rogers, Bassem Safadi, Paulina Salminen, Sergio Santoro, Nathaniel Sann, John D. Scott, Asim Shabbir, Stephanie Sogg, Erik Stenberg, Michel Suter, Antonio Torres, Surendra Ugale, Ramon Vilallonga, Cunchuan Wang, Rudolf Weiner, Natan Zundel, Luigi Angrisani, Maurizio De Luca

**Affiliations:** 1 Department of Surgery, Minimally Invasive Surgery Research Center, Division of Minimally Invasive and Bariatric Surgery, Hazrat-e Fatemeh Hospital, Iran University of Medical Sciences, Tehran, Iran; 2 Department of General and Laparoscopic Surgery, Obesity and Metabolic Surgery Unit, Ospedale Evangelico Betania, Naples, Italy; 3 Whittington Hospital, London, UK; 4 Department of Surgery, Center for Metabolic and Bariatric Surgery, Brigham and Women’s Hospital, and Harvard Medical School, Boston, MA USA; 5 Medical University of Vienna, Vienna, Austria; 6 Unitypoint Clinic Weight Loss Specialists, West Des Moines, IA USA; 7 Department of Surgery, University of Iowa, Iowa City, IA USA; 8 Bariatric Surgery Program, CHI Memorial Hospital, Chattanooga, TN USA; 9 Adelaide Bariatric Centre, Flinders University of South Australia, Adelaide, Australia; 10 Prisma Health, Department of Surgery, University of South Carolina School of Medicine, Greenville, SC USA; 11 WeightWorks Clinics and Allurion Clinics, Amersfoort, The Netherlands; 12 Iranian Hospital, Alwasl Road, Dubai, UAE; 13 Austin and Repatriation Medical Centre, University of Melbourne, Heidelberg, VIC Australia; 14 Department of General Surgery, Bariatric and Metabolic Institute, Cleveland Clinic, Cleveland, OH USA; 15 Minimally Invasive and Bariatric Surgery, Gastrointestinal Bariatric and Metabolic Center (GBMC)-Jordan Hospital, Amman, Jordan; 16 New Life Center, Guatemala, Guatemala; 17 Ventura Advanced Surgical Associates, Ventura, CA USA; 18 Centre of Excellence for the Study and Treatment of Obesity and Diabetes, Valladolid, Spain; 19 Department of Surgery, Texas Tech HSC Paul Foster School of Medicine, El Paso, TX USA; 20 Université Paris 5, Paris, France; 21 Center for the Treatment of Obesity and Diabetes, Hospital Alemão Oswaldo Cruz, Sao Paolo, Brazil; 22 Polyclinique Lyon Nord, Rillieux-la-Pape, France; 23 Department of General Surgery, AZ Sint Jan Brugge-Oostende, Bruges, Belgium; 24 Gastrocirurgia de Brasilia, University of Brasilia, Brasilia, Brazil; 25 Endovitta Institute, São Paulo, Brazil; 26 Division of Bariatric Surgery, CIUSSS-NIM, Montreal, Canada; 27 Department of Surgery, Université de Montréal, Montréal, Canada; 28 Bariatric Surgery Unit, Faculty of Medicine, Ain Shams University, Cairo, Egypt; 29 Makassed General Hospital, Beirut, Lebanon; 30 Fresno Heart and Surgical Hospital, UCSF Fresno, Fresno, CA USA; 31 Bariatric Surgery Unit, Delta Chirec Hospital, Brussels, Belgium; 32 University of Arizona College of Medicine, Phoenix, USA; 33 Department of Surgery, Massachusetts General Hospital, Boston, MA USA; 34 Weight Loss and Metabolic Surgery Center, Yotsuya Medical Cube, Tokyo, Japan; 35 Department of Digestive Surgery, CHU Félix Guyon, Saint Denis, La Réunion, France; 36 Walsall Healthcare NHS Trust, Walsall, UK; 37 Taiba Hospital, Kuwait City, Kuwait; 38 Digestive Disease and Surgery Institute, Cleveland Clinic, Cleveland, OH USA; 39 Department of Surgery, New York University Grossman School of Medicine, New York, NY USA; 40 Medical Weight Loss Center, China Medical University Shinchu Hospital, Zhubei City, Taiwan; 41 Director of St George Surgery, Sydney, Australia; 42 South Tyneside and Sunderland Foundation NHS Trust, Sunderland, UK; 43 University of Nebraska Medical Center, Omaha, USA; 44 NMC Royal Hospital, Abu Dhabi, UAE; 45 Bariatric Unit, Heraklion University Hospital, University of Crete, Crete, Greece; 46 Diakonissen Wehrle Private Hospital, Salzburg, Austria; 47 Cedars Sinai Medical Center, Los Angeles, USA; 48 Advanced Biomedical Sciences Department, Federico II” University, Naples, Italy; 49 BMI Surgery, New Lenox, IL USA; 50 Department of Nutrition and Dietetics, Leeds Teaching Hospitals, NHS Trust, Leeds, UK; 51 Division of Metabolic and Bariatric Surgery, Hartford Hospital, Hartford, CT USA; 52 Department of Surgery, Centro CIEN-Diagnomed, University of Buenos Aires, Buenos Aires, Argentina; 53 Department of General and Minimally Invasive Surgery, UT Health San Antonio, San Antonio, TX USA; 54 Department of Visceral Surgery, Clarunis, University Digestive Health Care Center, St. Clara Hospital and University Hospital Basel, Basel, Switzerland; 55 Department of Surgery Clinica Anglo Americana, Universidad Nacional Mayor de San Marcos, Lima, Peru; 56 Department of Surgery, Stanford School of Medicine, VA Palo Alto Health Care System, 3801 Miranda Avenue, GS 112, Palo Alto, CA 94304 USA; 57 New You Medical Center, King Saud University, Obesity Chair, Riyadh, Saudi Arabia; 58 Medical Director of Gastro-Obeso-Center, Institute for Metabolic Optimization, Sao Paulo, Brazil; 59 Department of Bariatric, Metabolic, and Plastic Surgery, St. Franziskus Hospital, Cologne, Germany; 60 Centro Multidisciplinar Do Tratamento da Obesidade, Hospital Lusíadas Amadora e Lisbon, Amadora, Portugal; 61 Department of Surgery - Division of Minimally Invasive and Bariatric Surgery, Penn State Health Milton S. Hershey Medical Center, Hershey, PA USA; 62 Aman Hospital, Doha, Qatar; 63 Division of Digestive Surgery and Urology, Department of Digestive Surgery, Turku University Hospital, Turku, Finland; 64 Hospital Israelita Albert Einstein, Av. Albert Einstein, 627, São Paulo, 05652-900 Brazil; 65 Advanced Surgical Partners of Virginia, Richmond, VA USA; 66 Division of Bariatric and Minimal Access Surgery, Department of Surgery, University of South Carolina School of Medicine, Greenville, SC USA; 67 National University of Singapore, Singapore, Singapore; 68 Massachusetts General Hospital Weight Center, Boston, MA USA; 69 Department of Surgery, Faculty of Medicine and Health, Örebro University, Örebro, Sweden; 70 Department of Surgery, Riviera-Chablais Hospital, Rennaz, Switzerland; 71 Department of Surgery, Hospital Clínico San Carlos, Complutense University of Madrid, Calle del Prof Martín Lagos, S/N, 28040 Madrid, Spain; 72 Kirloskar and Virinchi Hospitals, Hyderabad, Telangana India; 73 Endocrine, Bariatric, and Metabolic Surgery Department, Universitary Hospital Vall Hebron, Barcelona, Spain; 74 Department of Metabolic and Bariatric Surgery, The First Affiliated Hospital of Jinan University, Guangzhou, China; 75 Bariatric Surgery Unit, Sana Clinic Offenbach, Offenbach, Germany; 76 Department of Surgery, University of Buffalo, Buffalo, NY USA; 77 Department of Public Health, Federico II University of Naples, Naples, Italy; 78 Department of General Surgery Rovigo Hospital, Rovigo, Italy

**Keywords:** Procedure selection, Consensus, Metabolic surgery, Bariatric surgery, Class I and II obesity, Experimental models of disease, Obesity

## Abstract

Metabolic and bariatric surgery (MBS) is widely considered the most effective option for treating obesity, a chronic, relapsing, and progressive disease. Recently, the American Society of Metabolic and Bariatric Surgery (ASMBS) and the International Federation for the Surgery of Obesity and Metabolic Disorders (IFSO) issued new guidelines on the indications for MBS, which have superseded the previous 1991 National Institutes of Health guidelines. The aim of this study is to establish the first set of consensus guidelines for selecting procedures in Class I and II obesity, using an Expert Modified Delphi Method. In this study, 78 experienced bariatric surgeons from 32 countries participated in a two-round Modified Delphi consensus voting process. The threshold for consensus was set at an agreement or disagreement of ≥ 70.0% among the experts. The experts reached a consensus on 54 statements. The committee of experts reached a consensus that MBS is a cost-effective treatment option for Class II obesity and for patients with Class I obesity who have not achieved significant weight loss through non-surgical methods. MBS was also considered suitable for patients with Type 2 diabetes mellitus (T2DM) and a body mass index (BMI) of 30 kg/m^2^ or higher. The committee identified intra-gastric balloon (IGB) as a treatment option for patients with class I obesity and endoscopic sleeve gastroplasty (ESG) as an option for patients with class I and II obesity, as well as for patients with T2DM and a BMI of ≥ 30 kg/m^2^. Sleeve gastrectomy (1) and Roux-en-Y gastric bypass (RYGB) were also recognized as viable treatment options for these patient groups. The committee also agreed that one anastomosis gastric bypass (OAGB) is a suitable option for patients with Class II obesity and T2DM, regardless of the presence or severity of obesity-related medical problems. The recommendations for selecting procedures in Class I and II obesity, developed through an Expert Modified Delphi Consensus, suggest that the use of standard primary bariatric endoscopic (IGB, ESG) and surgical procedures (SG, RYGB, OAGB) are acceptable in these patient groups, as consensus was reached regarding these procedures. However, randomized controlled trials are still needed in Class I and II Obesity to identify the best treatment approach for these patients in the future.

## Introduction

Obesity is now recognized as a chronic disease associated with a pro-inflammatory state and is often undertreated. It has become a global epidemic, affecting populations worldwide^1,2^. Metabolic and bariatric surgery (MBS) is considered the most effective treatment option for obesity, which is a chronic, relapsing, and progressive disease^3^. These surgical interventions have been shown to induce both weight loss and remission of obesity-related medical problems. The recently released guidelines on the indications for MBS by the American Society of Metabolic and Bariatric Surgery (ASMBS) and the International Federation for the Surgery of Obesity and Metabolic Disorders (IFSO) in 2022^4^ have brought about changes to the previous beliefs based on the 1991 National Institutes of Health guidelines for bariatric surgery^5^.

The new guidelines suggest that MBS should be strongly recommended for patients with Class II obesity (BMI of 35–39.9 kg/m^2^) or higher, regardless of the presence or absence of obesity-related comorbidities. Additionally, MBS should be considered as a treatment option for individuals with Class I obesity (BMI of 30–34.9 kg/m^2^) who have obesity-related comorbidities such as type 2 diabetes mellitus (T2DM), hypertension (HTN), dyslipidemia (DLP), obstructive sleep apnea (OSA), gastroesophageal reflux disease (GERD), cardiovascular disease (CVD), non-alcoholic fatty liver disease and nonalcoholic steatohepatitis (NAFLD and NASH), chronic kidney disease (CKD), pseudotumor cerebri, asthma, polycystic ovarian syndrome (PCOS), infertility and musculoskeletal diseases that have not shown improvement with non-surgical treatment approaches (reviewer 2, comment 3).

New guidelines recommended modifying the BMI thresholds for the Asian population, wherein a BMI greater than 25 kg/m^2^ indicates clinical obesity and individuals with a BMI exceeding 27.5 kg/m^2^ should be provided with MBS as an option (reviewer 2, comment 1).

Although the new guidelines are evidence-based and supported by hundreds of published papers that recommend MBS for patients with Class I and II obesity, there are still numerous issues regarding the selection of the appropriate type of MBS, as well as technical details of the operation, for these groups of patients.

As such, the objective of this study is to develop the first consensus guidelines for procedure selection in Class I and II obesity utilizing an Expert Modified Delphi Method. The purpose of these guidelines is to provide clinicians with a useful tool for their daily clinical practice (reviewer 3, comment 2).

## Methods

The scientific core team of 12 members (Table 1) initiated the drafting of 70 statements with two choices (agree/disagree) and a comment box at the end of each statement, following a preliminary brainstorming session, literature review to find the issues with feedback from all the participants (reviewer 3, comment 4). The international consensus group, comprising distinguished academic and private surgeons, opinion makers in MBS, presidents of the ASMBS and IFSO, and renowned opinion leaders and multi-disciplinary team (MDT) members from all IFSO-chapters were invited to participate in a modified Delphi consensus-making exercise based on their prior MBS experience (reviewer 2, comment 4) and (reviewer 3, comment 3).

**Table 1:** List of the scientific core team (in alphabetical order).

Name	Country
*Luigi Angrisani*	Italy
*Sonja Chiappetta*	Italy
*Maurizio De Luca*	Italy
*Mohammad Kermansaravi*	Iran
*Shanu Kothari*	USA
*Lilian Kow*	Australia
*Teresa LaMasters*	USA
*Abdelrahman Nimeri*	USA
*Chetan Parmar*	UK
*Jaime Ponce*	USA
*Gerhard Prager*	Austria
*Scott Shikora*	USA

This consensus exercise was approved by the ethical committee of Iran University of Medical Sciences (Approval ID: IR.IUMS.REC.1400.361). The study included the participation of 78 renowned metabolic and bariatric surgeons from 32 countries, who constituted the Delphi consensus-building committee using an online platform (@Survey Monkey). The first round of consensus building commenced on April 7, 2023, and remained open until April 30, 2023. All committee members voted on the 70 statements, with only agree or disagree options. Agreement/disagreement levels ≥ 70.0% were considered as consensus. At the conclusion of the first round, 46 out of 70 statements achieved consensus. The scientific core team reviewed and merged several non-consensus statements based on the majority views of all voting experts, resulting in the finalization of 18 statements for the second round of voting to achieve a consensus on additional statements. The outcomes of the first round were communicated to all committee members, who were requested to vote on the remaining statements. The second round of consensus building commenced on May 18, 2023, and concluded on June 7, 2023.

### Ethical approval

All procedures performed in the study involving human participants were in accordance with the ethical standards of the institutional and/or national research committee and with the 1964 Helsinki declaration and its later amendments or comparable ethical standards. This consensus exercise was approved by the ethical committee of Iran University of Medical Sciences (Approval ID: IR.IUMS.REC.1400.361).

### Informed consent

Informed consent was obtained from the participants included in the consensus study.

## Results

A total of 78 experts in MBS from 32 countries participated in two rounds of voting to evaluate a series of statements. The detailed outcomes of the first and second rounds of voting for each statement are presented in Table 2. Among the 64 final statements, a consensus of at least 70% was attained for 54 statements, whereas the experts failed to reach a consensus for the remaining 10 statements even after two rounds of online voting.

**Table 2:**
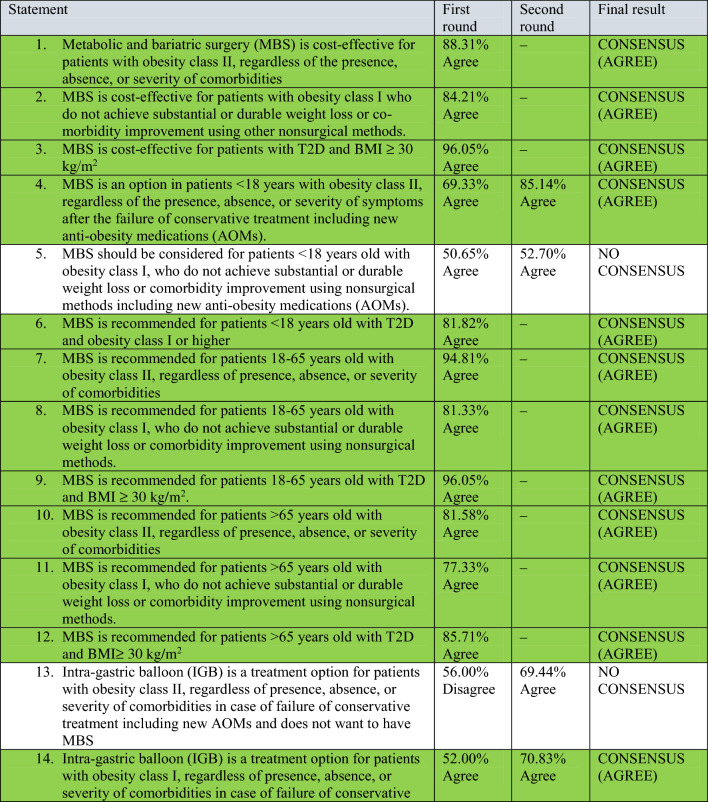

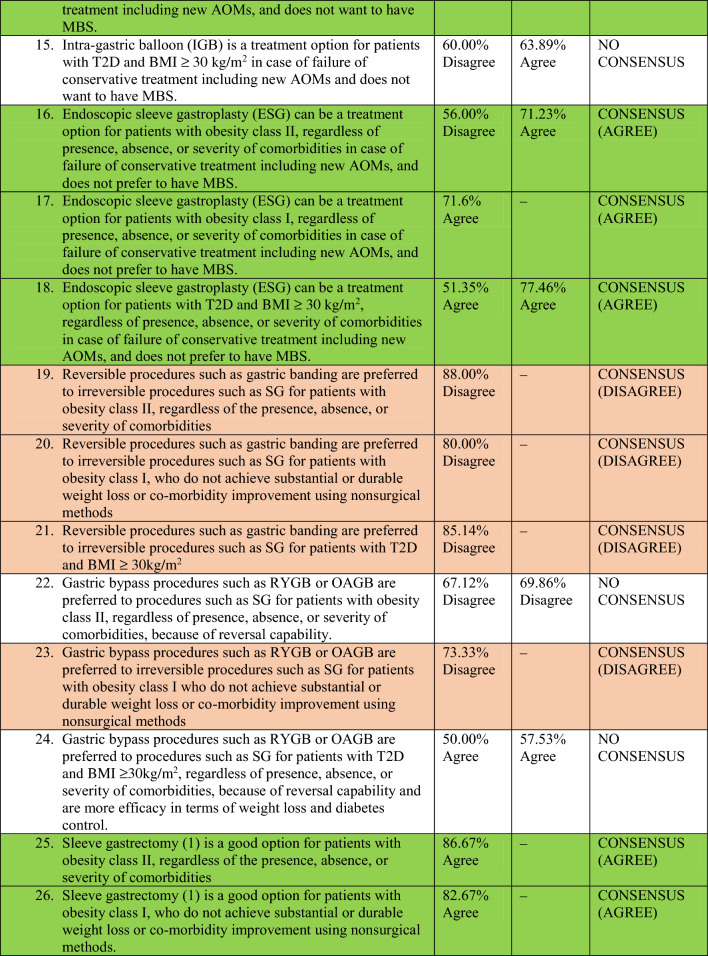

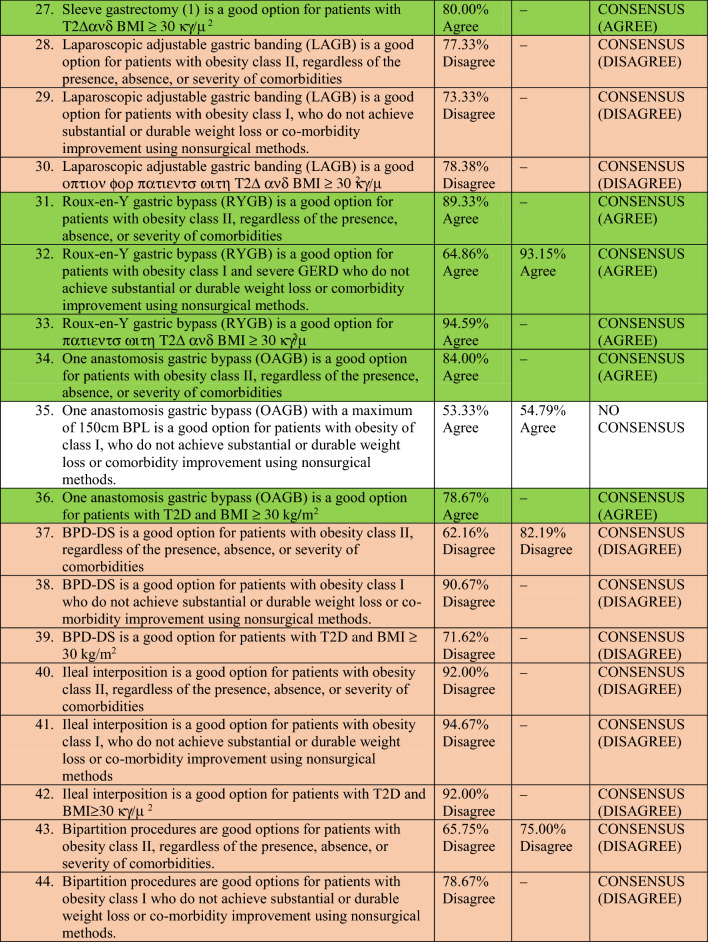

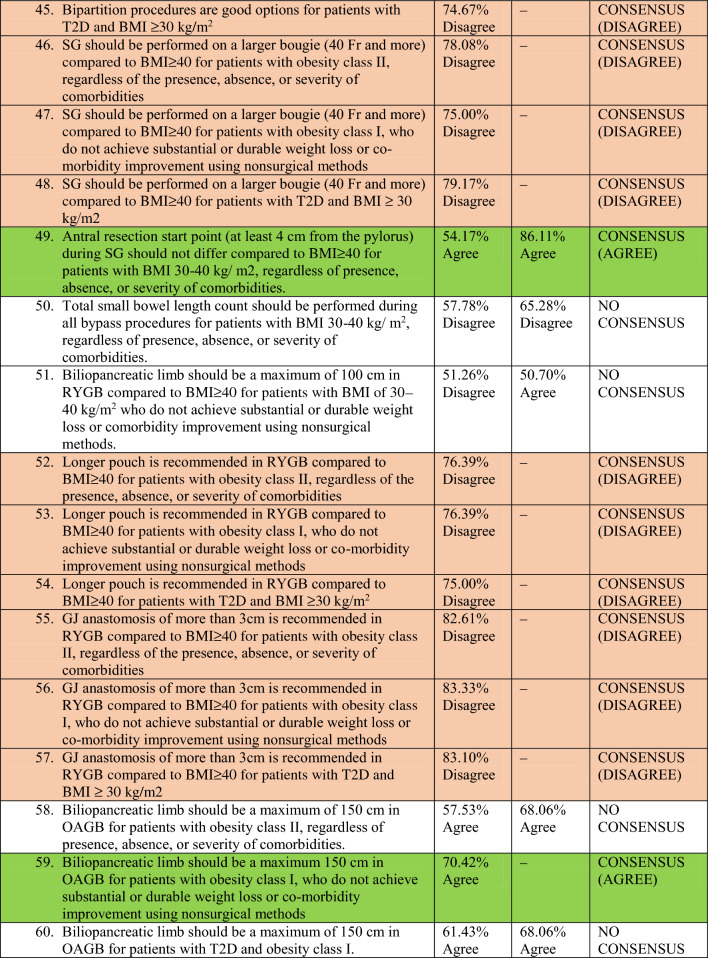

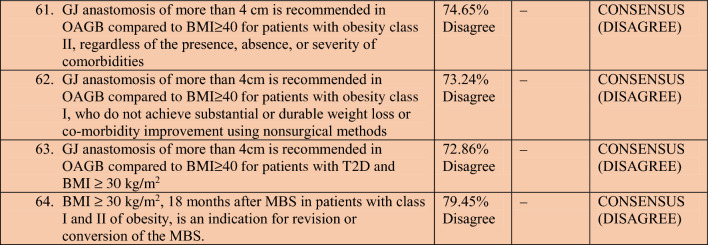
Consensus statements voting results.

### Cost effectiveness and indications of MBS (reviewer 3, comment 5)

According to the majority of experts, MBS is a cost-effective treatment option for patients with Class II obesity, regardless of the presence, severity, or absence of obesity-associated medical problems. Additionally, MBS is also considered suitable for patients with Class I obesity who fail to achieve significant or long-lasting weight loss or improvement in obesity-associated medical problems through non-surgical methods, as well as for patients with Type 2 Diabetes Mellitus (T2DM) and a BMI of 30 kg/m^2^ or higher. As mentioned above, BMI thresholds is lower in Asian, and the patients with BMI > 27.5 kg/m^2^ should be offered MBS (reviewer 2, comment 1).

### MBS in the extreme of ages

The majority of experts concurred that MBS can be considered as a treatment option for patients below 18 years of age who have Class II obesity, irrespective of the presence, severity, or absence of symptoms, especially after conservative treatment options such as new Anti-obesity medications (AOMs) have failed. MBS is also considered an option for patients under 18 years of age who have T2DM and Class I or higher obesity. However, there was no consensus among experts regarding the use of MBS for patients below 18 years of age who have Class I obesity and fail to achieve substantial or long-lasting weight loss or obesity-associated medical problems improvement using non-surgical methods.

### Primary endoscopic bariatric procedures

There was a consensus on the use of intra-gastric balloon (IGB) as a treatment option for patients with Class I obesity, irrespective of the presence, absence, or severity of obesity-associated medical problems, particularly when conservative treatment options including new AOMs have failed, and the patient is unwilling to undergo MBS. However, there was no consensus on the use of IGB as a treatment option for patients with Class II obesity or patients with T2DM and a BMI of ≥ 30 kg/m^2^, in case of failure of conservative treatment.

The majority of participants expressed the view that endoscopic sleeve gastroplasty (ESG) can be considered as a treatment option for patients with Class I and II obesity, as well as patients with T2DM and a BMI of ≥ 30 kg/m^2^, regardless of the presence, absence, or severity of obesity-associated medical problems, particularly when conservative treatment options, including new AOMs, have failed, and the patient is unwilling to undergo MBS.

### Metabolic and bariatric surgical procedure selection

There was disagreement consensus on adjustable gastric banding (AGB) as a treatment option for patients with Class I and II obesity, as well as patients with T2DM and a BMI of ≥ 30 kg/m^2^.

Conversely, there was a consensus agreement on Sleeve Gastrectomy^6^ and Roux-en-Y Gastric Bypass (RYGB) as viable treatment options for the three aforementioned groups of patients. There was also a disagreement consensus on the suitability of Biliopancreatic Diversion- Duodenal Switch (BPD-DS), Ileal Interposition, and bipartition procedures as treatment options for all the three aforementioned groups of patients.

According to the majority of experts, one anastomosis gastric bypass (OAGB) is a suitable treatment option for patients with Class II obesity, irrespective of the presence, absence, or severity of obesity-associated medical problems, as well as for patients with T2DM and a BMI of ≥ 30 kg/m^2^. However, there was no consensus among experts regarding the use of OAGB for patients with Class I obesity who fail to achieve substantial or long-lasting weight loss or comorbidity improvement using non-surgical methods.

### Technical aspects of three common MBS procedures (reviewer 3, comment 7)

#### Sleeve gastrectomy (SG)

Most of the participants were in disagreement about the use of larger bougies (40 Fr and above) for SG in patients with Class I and II obesity as well as patients with T2DM and a BMI of ≥ 30 kg/m^2^ (disagreement consensus).

As per the majority of experts, the starting point for antral resection during SG should not vary for patients with a BMI of 30–40 kg/m^2^ compared to those with a BMI of ≥ 40 kg/m^2^. They concluded that antral resection should be initiated at a distance of at least 4 cm from the pylorus, regardless of the presence, absence, or severity of comorbidities.

#### Roux-en-Y gastric bypass (RYGB)

There was disagreement consensus on whether to create a longer pouch or a gastro-jejunal anastomosis exceeding 3 cm in patients with Class I and II obesity, or patients with T2DM and a BMI of ≥ 30 kg/m^2^, as compared to patients with higher BMI (BMI ≥ 40 kg/m^2^).

There was no consensus on the optimal length of the Biliopancreatic Limb (BPL) of RYGB for patients with a BMI of 30–40 kg/m^2^ compared to those with a BMI of ≥ 40 kg/m^2^.

#### One anastomosis gastric bypass (OAGB)

Most experts believed that the BPL of OAGB should not exceed 150 cm for patients with Class I obesity. However, there was no consensus on the optimal length of the BPL of OAGB for patients with Class II obesity or patients with T2DM and Class I obesity. There was disagreement consensus among the majority of experts regarding the creation of a gastro-jejunal anastomosis exceeding 4 cm during OAGB for patients with Class I and II obesity, or patients with T2DM and a BMI of ≥ 30 kg/m^2^, as compared to patients with higher BMI (BMI ≥ 40 kg/m^2^).

## Discussion

### Cost effectiveness of MBS

There is enough literature to prove that MBS is cost-effective for the treatment of obesity^7^. It has also been shown to give better long-term results compared to intensive medical therapy for patients with T2DM and BMI between 27 and 43^8^. Recently the updated IFSO-ASMBS guidelines have reduced the BMI criteria for eligibility for MBS^4^. Hence it was no surprise that the experts agreed to a consensus that MBS is cost-effective for patients with class II obesity regardless of comorbidities and class I obesity with T2DM and also those who do not achieve weight loss outcomes with non-surgical methods.

### MBS in the extreme of ages

Long-term follow-up after LSG in a prospective study of 2504 children and adolescents with class II/III obesity demonstrates durable weight loss, maintained comorbidity resolution, and unaltered growth^9^ It is also shown that it is important to tackle obesity during childhood with MBS if possible before complications ensue later in life^10^. The experts also agreed that MBS should be considered in patients < 18 years with class II obesity regardless of comorbidities which reflects the published literature. Since no consensus in patients with class I obesity and age < 18 years was reached, these patients should be treated with caution and detailed individual decisions in an interdisciplinary team might be the only way to approach obesity treatment in these patients.

### Procedure selection

#### Primary endoscopic bariatric procedures

IGB and ESG are two primary endoscopic bariatric interventions. There are currently three FDA-approved gastric balloon devices: Orbera balloon, Obalon balloon, and Spatz3 balloon. Apollo Endosurgery received FDA approval for Apollo ESG and Apollo REVISE Systems on July 12, 2022. Endoscopic bariatric procedures are low-risk procedures and are applicable to the population who are not candidates for MBS^11^. Expert consensus for the use of IGB in Obesity Class I and the use of ESG in Class I, II and as a metabolic treatment option in patients with T2DM was achieved in this Expert Modified Delphi Consensus, reflecting the actual trend and rise of endoscopic bariatric procedures, especially in Class I and II Obesity.

Actual evidence shows that IGB is more effective than lifestyle intervention alone with a reported difference in mean in %EWL and %TWL at follow-up of 17.98%, and 4.40% in a systematic review and meta-analysis including thirteen RCTs with 1523 patients^12^. Efficacy and safety of ESG were reported in a systematic review and meta-analysis including 8 original studies and 1772 patients, with pooled post-ESG rate of severe adverse events of 2.2% and a mean TBWL of 16.5% and 17.2% at 12 and 18–24 months^13^. Consensus is therefore in line with clinical practice and scientific evidence and the use of primary endoscopic bariatric procedures might be an important approach in patients with Class I and II Obesity.

#### Adjustable gastric banding (AGB)

Disagree Consensus for the use of gastric banding in Class I and II Obesity and superiority of AGB over SG was achieved both in the first Delphi round. The history of AGB, the use of a foreign body, and the complications in the long term all explain this disagreeing consensus. A systematic review performed in 2015 concluded that the role of LAGB in bariatric surgery is worthy of further appraisal, by comparing it with other types of bariatric procedures, because of the limited high-quality evidence^14^. Finally, the fall in the number of AGB performed in recent years (1.4% in 2018)^15^ underlines that it is a rarely performed procedure nowadays and therefore it’s reduced its role in Class I and II obesity.

#### Roux-en-Y gastric bypass and one anastomosis gastric bypass (OAGB)

This expert consensus disagrees that RYGB procedures are preferred to SG in Class I (disagree consensus) and Class II Obesity (disagree 69.86%). This might be based on the fact, that the perception of long-term complications seems to be higher after gastric bypass procedures compared to SG. Nevertheless, the SLEEVEPASS trial with a 10-year follow-up showed no statistically significant differences in either long-term complication rates or remission of T2DM, dyslipidemia, or obstructive sleep apnea (OSA) between the procedures^16^. SM-BOSS trial showed at a 5-year follow-up, that even if the type of long-term complications is different, the frequency is not statistically different^17^. Since both SG and RYGB resulted in good and sustainable weight loss^16,17^, it might be understandable, why gastric bypass procedures are not preferred to SG. On the other hand, agreement consensus was achieved for RYGB being a good option in patients with Class II Obesity, Class I Obesity and severe GERD, and patients with T2DM and Class I/II Obesity. Agreement consensus was achieved for OAGB being a good option in patients with Class II Obesity and patients with T2DM and a BMI of ≥ 30 kg/m^2^. This statement reflects that RYGB and OAGB are accepted procedures in Class II Obesity and also accepted metabolic bariatric procedures. This is in congruence with the current literature, since RYGB might be the surgical procedure of choice in GERD after primary bariatric procedures and primary anti-reflux procedures^18,19^ and might also be superior to anti-reflux procedures as a primary indication^20,21^. Nevertheless, the role of duodenal exclusion in the treatment of T2DM is well known^22^.

A recent network meta-analysis Expert Panel and Evidence Review Team for the Italian Guidelines on Bariatric and Metabolic Surgery analyzed different types of MBS with non-surgical therapy for the treatment of T2DM. The meta-analysis showed that MBS was associated with a significantly higher reduction of HbA1c, T2DM remission, and BMI compared with medical therapy and a significant reduction of HbA1c was observed with OAGB and SG and in addition, RYGB and OAGB were associated with a significant reduction of BMI. The group concluded therefore, that MBS is an effective option for the treatment of T2DM in patients with obesity, but pointed out that further long-term trials of appropriate quality are needed for assessing the risk–benefit ratio in some patient cohorts, such as those with a BMI of less than 35 kg/m^2^^23^.

#### Sleeve gastrectomy

Agreement consensus was achieved for SG being a good option in patients with Class I and II obesity, and patients with T2DM and a BMI of 30 kg/m^2^ or higher. The consensus shows, that SG and RYGB are both equally accepted procedures, without inferiority of one procedure^16,17^ and therefore next to the Expert Consensus both applicable to Class I and II obesity.

#### BPD-DS, ileal interposition

Both procedures, BPD-DS, and ileal interposition, achieved a complete disagree consensus in nine statements regarding the usage in Class I/II Obesity. Since ileal interposition is not standard primary care in MBS^24^ and long-term complications, including predominantly malnutrition, are too high after BPD-DS^25^, both procedures should not be performed in patients with Class I/II Obesity.

### Technical issues

In a survey by Gagner M et al. they reported that majority (40%) of the surgeons prefer 36F bougie for calibration of their SG (Range 32-50F)^26^. Hence, it was not surprising that there was a consensus in disagreement that a bougie of 40F or more should be used for calibration in patients with Classes I and II obesity during SG. The argument for suggesting that recommendation was that a wider pouch would be safe in these low BMI patients to avoid patients from getting malnourished.

There is an ongoing debate about the ideal distance from the pylorus for the first staple firing in the case of SG. There is some evidence that antral resection gives better weight loss outcomes compared to where it is preserved^27^. There is a recent RCT that has shown that firing the stapler 2 cm from the pylorus gives better weight loss outcomes and also better control of T2DM^28^. However, in our Delphi consensus there was an agreement that the start point should be at least 4 cm from the pylorus.

There was no consensus to say that total bowel length was counted during all bypass procedures even in patients with Classes I and II Obesity. Similarly, there was no consensus that the BPL should be a maximum of 100 cm in RYGB in these patients. There is no consensus at the moment about ideal BPL and measuring all small bowel lengths as there are risks and advantages for the same^29^. There have been some malnutrition issues reported after longer BPL in cases of OAGB^30,31^. The experts agreed in consensus that 150 cm should be the maximum BPL in patients with Class I Obesity^6^. However, they disagreed that should be the case in patients with class II obesity and also in those with class I and T2DM suggesting that it could be tailored according to the BMI^6,32^.

There have been some reports that making a longer gastric pouch in the case of RYGB gives better weight loss outcomes and reduces weight regain^33,34^. However, the experts disagreed in consensus that the length of the gastric pouch or the size of the anastomosis (> 3 cm) should not be increased in this cohort of patients in the case of RYGB. Similarly, they disagreed that the GJ anastomosis should be more than 4 cm in this cohort of patients regardless of their BMI^6,32,35^.

There is no clearly defined break point for indication for revision or conversion of the MBS in case of poor weight loss or weight regain. Interestingly the experts disagreed that BM > 30 kg/m^2^ should be an indication for revisional surgery after 18 months of the primary procedure.

## Conclusion

This current recommendations for procedure selection in Class I and II Obesity developed through an Expert Modified Delphi Consensus, conclude that the use of the standard primary bariatric endoscopic (IGB, ESG) and surgical procedures (SG, RYGB, OAGB) is also accepted in Class I and II Obesity since statements reached consensus regarding these current procedures. Nevertheless, randomized controlled trials in this patient’s class are still necessary to give our patients with Class I and II Obesity the best treatment approach in the future.

## Data Availability

The datasets used and/or analyzed during the current survey available from the corresponding author on reasonable request.
